# Utilizing Gene Tree Variation to Identify Candidate Effector Genes in *Zymoseptoria tritici*

**DOI:** 10.1534/g3.115.025197

**Published:** 2016-01-29

**Authors:** Megan C. McDonald, Lachlan McGinness, James K. Hane, Angela H. Williams, Andrew Milgate, Peter S. Solomon

**Affiliations:** * Plant Sciences Division, Research School of Biology, The Australian National University, Canberra, ACT, Australia 2601; † Black Box Bioinformatics, Perth, WA, Australia 6102; ‡ CCDM Bioinformatics, Centre for Crop and Disease Management, Department of Environment and Agriculture, Curtin University, Perth, WA, Australia; § Curtin Institute for Computation, Curtin University, Perth, WA, Australia 6102; ** NSW Department of Primary Industries, Wagga Wagga Agricultural Institute, NSW, Australia 2795

**Keywords:** *Mycosphaerella graminicola*, comparative genomics, intraspecific, fungal effector, accessory chromosome

## Abstract

*Zymoseptoria tritici* is a host-specific, necrotrophic pathogen of wheat. Infection by *Z. tritici* is characterized by its extended latent period, which typically lasts 2 wks, and is followed by extensive host cell death, and rapid proliferation of fungal biomass. This work characterizes the level of genomic variation in 13 isolates, for which we have measured virulence on 11 wheat cultivars with differential resistance genes. Between the reference isolate, IPO323, and the 13 Australian isolates we identified over 800,000 single nucleotide polymorphisms, of which ∼10% had an effect on the coding regions of the genome. Furthermore, we identified over 1700 probable presence/absence polymorphisms in genes across the Australian isolates using *de novo* assembly. Finally, we developed a gene tree sorting method that quickly identifies groups of isolates within a single gene alignment whose sequence haplotypes correspond with virulence scores on a single wheat cultivar. Using this method, we have identified < 100 candidate effector genes whose gene sequence correlates with virulence toward a wheat cultivar carrying a major resistance gene.

*Zymoseptoria tritici* is a fungal wheat pathogen responsible for the most serious disease of wheat in the United Kingdom, and other parts of Northern Europe (te Beest *et al.* 2013; Dean *et al.* 2012; Fones and Gurr 2015). The disease is characterized by a long latent period, typically lasting 8–11 d before the first appearance of visible necrotic lesions (Sánchez-Vallet *et al.* 2015). While historically considered a “hemibiotroph,” there is gathering evidence from metabolomics, microarray, and RNA sequencing studies that *Z. tritici* does not feed from living host cells, as per a biotroph, at any stage during its lifecycle (Keon *et al.* 2007, 2005; Rudd *et al.* 2015). In light of this evidence, it has been proposed that *Z. tritici* be reclassified as a “latent necrotroph” (Sánchez-Vallet *et al.* 2015). *Z. tritici* penetrates its host through open stomata and grows sparsely in the intercellular space for the duration of the latent period. This early growth is followed by rapid host cell death, and proliferation of hyphae throughout the host tissue (Kema *et al.* 1996). Asexual sporulation occurs almost exclusively in the substomatal cavities (Cohen and Eyal 1993; Kema *et al.* 1996). The close and prolonged relationship with the plant during early infection suggests that *Z. tritici* possesses a sophisticated, and strictly regulated, set of proteins or metabolites that are responsible for restricting its growth in the host, suppressing plant recognition/immunity, and ultimately inducing host-cell death. These proteins or metabolites are commonly referred to as effectors (de Wit *et al.* 2009; Stergiopoulos and de Wit 2009).

Identification of pathogen effectors (and their corresponding host targets) is essential for understanding how these parasites are able to successfully coopt plants into productive food sources. This is especially true in agricultural ecosystems, where host genetic uniformity provides ample opportunity for specialists to emerge (Stukenbrock and Bataillon 2012; Stukenbrock and McDonald 2008). Often, it is the effector genes that are responsible for host- or cultivar-driven specificity (de Wit *et al.* 2009; Stergiopoulos and de Wit 2009). Many described effectors interact with specific resistance or susceptibility genes in their hosts. These specific interactions are referred to as “gene-for-gene” relationships (Dodds and Rathjen 2010). Despite large differences in pathogen lifestyles and host range, several common characteristics are shared between these genes. Generally, proteinaceous fungal effectors are considered to be small (< 300 aa), secreted, cysteine-rich (> 3%, after signal peptide cleavage), and induced during *in planta* infection (de Wit *et al.* 2009; Hogenhout *et al.* 2009). These characteristics are broadly used to prioritize effector gene candidates for functional studies, though recent work has shown that these characteristics do not encompass all known effectors (Sperschneider *et al.* 2015).

In filamentous fungi, effector genes are also commonly found in association with rapidly evolving segments of the genome, such as repeat-rich regions, or on accessory chromosomes (ACs). For example, *AvrPita* in *Magnaporthe oryzae*, *SIX* genes in *Fusarium oxysporum*, and the PEP cluster in *Nectria hematococca*, are all located on ACs (Chuma *et al.* 2011; Rodriguez-Carres *et al.* 2008; Schmidt *et al.* 2013). *Z. tritici* has several ACs that are well described, though, unlike other fungal pathogens, they have never been associated with pathogenicity (Croll and McDonald 2011; Croll *et al.* 2013; Stukenbrock *et al.* 2007; Wittenberg *et al.* 2009). Other well-characterized necrotrophic effectors, such as ToxA in *Pyrenophora tritici-repentis*, and Tox3 and Tox1 in *Parastagonospora nodorum*, were successfully identified using culture filtrates that induced necrosis when infiltrated into susceptible wheat varieties (Liu *et al.* 2009, 2012; Tuori *et al.* 1995). This approach has recently identified two necrosis inducing proteins, ZtNIP1 and ZtNIP2, in *Z. tritici* (Ben M’barek *et al.* 2015). Heterologous expression and infiltration of these proteins into wheat also revealed cultivar specificity, though it remains unclear if this is due to a gene-for-gene, or a more general necrosis inducing response (Ben M’barek *et al.* 2015). Several in-depth RNA-sequencing (RNA-seq) studies with *Z. tritici* have identified many highly expressed “effector-like” genes or secondary metabolite clusters; however, no effector genes critical for virulence were identified in these studies (Kellner *et al.* 2014; Rudd *et al.* 2015).

Thus far, the only gene that has been shown to be essential for virulence in *Z. tritici* is *Mg3LysM*, which was discovered based on close homology to another previously described effector gene *Ecp6* (Marshall *et al.* 2011). Three additional small secreted proteins (SSPs) that contribute quantitatively to virulence were recently described by Poppe *et al.* (2015). These genes were selected for functional analysis because they exhibited positive (syn. diversifying) selection (dN/dS > 1), when compared to genomes of nonwheat-infecting relatives *Zymoseptoria pseudotritici* and *Zymoseptoria ardabillae* (Poppe *et al.* 2015; Stukenbrock *et al.* 2011). Here, the authors showed that the *Z. tritici* orthologs contributed more to virulence on wheat than the corresponding ortholog from the nonwheat-infecting relatives. Despite the emerging consensus that this disease is highly quantitative in nature, at least one gene-for-gene interaction with the qualitative resistance gene *Stb6* has been demonstrated genetically, and several major resistance genes in wheat have been mapped (Brading *et al.* 2002; Orton *et al.* 2011; Stewart and McDonald 2014).

With the advent of next generation sequencing, whole-genome comparisons of fungal pathogens are now routine (Thynne *et al.* 2015). Many studies focus on comparative analysis between closely related species with varying host ranges or pathogenic lifestyles (Gardiner *et al.* 2012; Manning *et al.* 2013; Stukenbrock *et al.* 2011). These studies provide significant insight into how pathogenic fungi are able to occupy similar niches, *i.e.*, host specialization (Gardiner *et al.* 2012). Yet, so far, intraspecific resequencing of multiple isolates has been limited to a small number of key pathogen species (Chen *et al.* 2013; Persoons *et al.* 2014; Xue *et al.* 2012). Comparative genome studies with *Z. tritici* are aided by the completeness of the reference genome, Dutch isolate IPO323 (Goodwin *et al.* 2011). Stukenbrock *et al.* (2011) sequenced two additional *Z. tritici* genomes from Iran, and several genomes of closely related relatives to examine interspecific changes that may lead to specialization on wheat. This analysis showed accelerated evolution specifically in the *Z. tritici* species lineage when compared to closely related relatives. Torriani *et al.* (2011) added an additional seven *Z. tritici* genomes from Switzerland to examine intron presence/absence (P/A) polymorphisms, both within and between species. Croll *et al.* (2013) used these same seven Swiss isolates, plus an additional four newly sequenced genomes, to describe the process of extensive genome rearrangement in ACs. Each of these studies reinforces the perception of *Z. tritici* as a highly dynamic, diverse pathogen, with the potential to rapidly evolve at the gene or genomic level within a single sexual generation (Croll *et al.* 2013).

In Australia, the breaking of the Millennium drought (2001–2009) has driven the reemergence of *Z. tritici* as a damaging pathogen of wheat in the high-rainfall areas of Victoria and South Australia (Hollaway 2014; Hollaway and McLean 2014; Milgate *et al.* 2015, 2014). Little is known about the current state of the Australian *Z. tritici* populations, particularly in terms of which wheat resistance genes remain effective at controlling the disease. In this study we characterized the virulence of 13 *Z. tritici* isolates, collected over 30 yr from all major wheat-producing regions of Australia. Virulence scores were measured on a differential set of 11 wheat cultivars, many of which contain known resistance genes. Subsequently, we sequenced the genomes of each *Z. tritici* strain in order to assess whether genetic variation, particularly within coding regions, could be associated with observed virulence profiles.

## Materials and Methods

### Growth of fungal strains and pathogenicity assays

The protocol for media and inoculum preparation was followed as described previously (Ballantyne and Thomson 1995). Plants were grown, inoculated and assessed as previously described in Zwart *et al.* (2010) at Wagga Wagga, NSW, with the following modifications. Four seedlings of each differential genotype were grown in a pot, and treated as an experimental unit with two replications per experiment. The full list of fungal isolates and cultivars used is provided in Table 1.

**Table 1:** Pathogenicity scores on 11 differential wheat cultivars

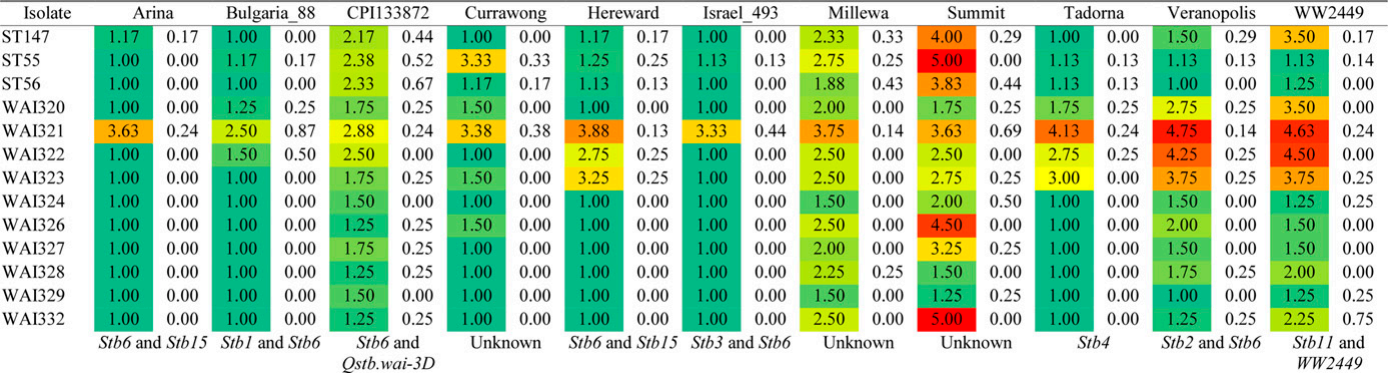

Isolate virulence is scored visually on a quantitative scale from 1 to 5, with 5 being highly virulent. Mean scores are colored to highlight differences in virulence across cultivars [1 = green (minimum), 3 = yellow (midpoint), and 5 = red (maximum)]. Standard errors for each disease score are given in the following column (white). Below each cultivar column is the postulated Septoria tritici *blotch* (*Stb*) resistance gene present in that cultivar based on previous publications (Chartrain *et al.* 2005; Arraiano and Brown 2006; Goodwin 2007; Raman *et al.* 2009; Zwart *et al.* 2010; Brown *et al.* 2015). Columns with “unknown” indicate that the locus/loci responsible for resistance has yet to be characterized.

After all disease scores were assigned, the average score was used to group the isolates based on their virulence profile in two independent ways. First, by calculating the pairwise Euclidean distance between each isolate’s virulence profile using the dist() function in R. This distance matrix was then used to cluster isolates using hclust() with Ward’s method, which clusters by minimizing the sums of squares between isolates. The resulting dendrogram was plotted in R using the plot() function. The same data were used to cluster the isolates using a K-means method, also implemented through R. Unlike the clustering method described above, where a dendrogram is generated to visualize any potential clusters, K-means requires selection of the ideal number of clusters *a priori*. K-means centers were plotted with three and four clusters on top of the raw virulence scores for each isolate on the cultivars Hereward (*x*-axis) and Summit (*y*-axis). From this plot, the fit of k-means centers to the data was visually assessed, and the grouping compared to that derived from the distance-based method described above.

### DNA extraction and sequencing

Fungal yeast-like spores were grown, as described above, from –80 **°**C glycerol stocks. For DNA extraction, harvested spores were concentrated into pellets via centrifugation. These pellets were cooled to –80° and lyophilized for 24 hr. DNA was extracted using the QIAGEN Plant mini-prep kit (QIAGEN, VIC, Australia) following the standard protocol. Extracted DNA was submitted to the Australian Cancer Research Foundation (ACRF) Biomolecular Resource Facility (JCSMR, Australian National University). Here, libraries were constructed using the Illumina TruSeq DNA LT Sample Prep kit v2 (part # FC-121-2001). Samples WAI320–WAI329 were multiplexed and sequenced together at 100 bp paired end using a v3 Illumina TruSeq SBS Kit (HS 200 cycles, part # FC-401-3001) in a single lane on a HiSeq2000 at the ACRF. Samples WAI55, WAI56, WAI147, and WAI332 were sequenced earlier at the Australian Genome Research Facility (Melbourne, VIC). Raw reads were trimmed of any remaining adapters using Trimmomatic v0.27 (specific Trimmomatic options: ILLUMINACLIP: TruSeq2_PE.fa:2:30:10 LEADING:20 TRAILING:20 SLIDINGWINDOW:4:24 MINLEN:90) (Bolger *et al.* 2014). All sequenced samples were checked for quality using FastQC v0.10.1 (http://www.bioinformatics.babraham.ac.uk/projects/fastqc/). Paired reads are deposited in the NCBI short-read archive: Bioproject ID PRJNA299857 and Biosample IDs SAMN04216882-SAMN04216894. Isolates were assembled *de novo* using the SPAdes Genome Assembler v3.5 as described previously (Bankevich *et al.* 2012; McDonald *et al.* 2015).

### Mappability of the reference genome IPO323

Mappability is defined as the inherent ability of regions within a genome to be correctly mapped using short-sequencing read technology. Many areas of the genome, including coding regions, may not be reliably mapped with short length reads. This will influence both the reliability and accuracy of single nucleotide polymorphisms (SNPs) called in low mappability regions. We calculated mappability on the reference genome isolate IPO323 using a modified version of the program gmav0.1.3 (Lee and Schatz 2012) (https://github.com/cbergman/gma-0.1.3), and excluded regions of low mappability from variant call format (VCF) files after SNP calling (defined here as a mappability score < 50). The mappability score is influenced by the sequencing technology; therefore, for this project, we used the settings (tech–illumina -b 50).

### Sequence variation in resequenced genomes

#### SNP calling with the genome analysis toolkit:

Trimmed paired and unpaired reads were mapped to the reference genome IPO323 v2.0 (http://genome.jgi.doe.gov/Mycgr3/Mycgr3.home.html) using BWAv0.7.3a-r367. Duplicate reads within the bam files were removed with the Picard v1.87 tool Mark Duplicates, and then labeled with the date of the sequencing run with the Genome Analysis Toolkit (GATK) tool AddOrReplaceReadGroups. The bam files were then used as input for the GATK variant call pipeline using the following tools, respectively, RealignerTargetCreator, IndelRealigner and HaplotypeCaller. Final SNP calls were filtered using VariantFiltration with the following options: -R /LargeDataSet/Megan/GATK/Mycosphaerella_graminicola.allmasked.fa-o Ztritici.filteredSNPS.GATK3-30–filterExpression “QD, 2.0 || FS > 50.0 || MQ, 25.0”–filterName “haploidfilter” -mask mappability_51_0.5andless_3col.bed -maskName “lowmappability”. These variants were then removed using an inverse grep bash command (grep –v “low mappability”). The original prefiltered VCF file is available as Supporting Information File S1. The bed file used to mask the VCF file is provided as File S2. The filtered VCF file was then further annotated with SNPeffv3.2a (Cingolani *et al.* 2012). To limit the assignment of multiple effects to SNPs with close neighboring genes, we reduced the up and downstream region of the genes to 500 bp using the –ud option (default 5 kb). The genome annotation used for SNP annotation is the RNA-seq based annotation released by Rothamsted Research on Ensembl Fungi (http://fungi.ensembl.org/Zymoseptoria_tritici/Info/Index, gff files available on request from Dr. Jason Rudd at Rothamsted Research, UK). Blast2GO v3.1.2 was used to assign gene ontolotgy (GO) terms and ProtIDs to this annotation. Signal peptide annotations were predicted SignalP v4.1 (Emanuelsson *et al.* 2007).

#### Gene presence/absence:

BLASTN searches using each *de novo* assembly of the Australian isolates as the BLAST database, and IPO323 genomic DNA coding regions as the BLAST query were conducted as described by McDonald *et al.* (2015). Briefly, results were limited to the top BLAST hit (maximum bit score), with an e-value cut-off of 0.0001. The regions for each BLAST hit were extracted from each of the *de novo* assemblies, and written to a combined FASTA file. These FASTA files were aligned using MUSCLEv3.8.31 (Edgar 2004). Gene presence was called if the top BLAST hit length was ≥ 75% of the annotated gene in IPO323.

### Phylogenetic analyses

A phylogenetic tree was constructed using the Bayesian Markov Chain Monte Carlo (MCMC) model implemented in BEASTv2.3.0 (Bouckaert *et al.* 2014). A total of 905 gene alignments that contained all 13 resequenced isolates were selected at random, and concatenated as input for Beast (Total alignment length 1,534,110 bp). Beauti v2.30 was used to format the XML file that specifies the model parameters to be run in BEAST (Bouckaert *et al.* 2014). Runs were conducted assuming the General Time Reversible nucleotide substitution model with no rate variation categories, a constant Population size, and a strict molecular clock. After a burn-in of 100,000 steps, 10,000 posterior trees were sampled. To assess run convergence, the BEAST log file was visualized in Tracer v1.6.0. The estimated sample size for each parameter was well over 100, which indicates that sampling of the posterior space during the run was sufficient. The sampled 10,000 posterior trees were visualized using Densi Tree v2.2.2 (Bouckaert and Heled 2014). This procedure was repeated independently two times with a smaller set of 500 randomly selected genes, to ensure that gene selection did not affect the final tree topology (data not shown). To verify that concatenation did not adversely affect our tree construction, we used the whole genome phylogenetic software andi (Haubold *et al.* 2015). The entire *de novo* assembly FASTA files for each isolate were used as input for this analysis. The resulting distance matrix was plotted as a tree using R. Haplotype networks were drawn with TCS v1.21 implemented in the software popart (http://popart.otago.ac.nz) (Clement *et al.* 2002).

### Matching phenotype to individual gene trees

In order to quickly sort through gene trees, and search for specific grouping of isolates based on phenotype, we created PhyBi, written in python (https://github.com/SolomonLab/PhyBi). PhyBi creates a phylogenetic “binary tree” for each gene by grouping isolates based on their sequence similarity. First, a matrix is created by parsing a Newick formatted tree to calculate pairwise distances (created by summing the branch lengths) between any two isolate pairs (referred to as the “distance matrix”). By definition, the distance matrix is symmetric, and all diagonal entries are zero (distance between an isolate and itself). If each of these distances is below a given threshold (default 0.01 substitutions per site), all isolates are grouped together and a “star” result is recorded. This will collapse genes whose sequence is near identical (< 1 bp substitution per 100 bp) into a single group and not consider them further. In cases where isolates differ by more than the first threshold, a second threshold is used to move the isolates into groups. If any two isolates differ by less than the second threshold (default 0.02 substitutions per site), they are designated to the same group. This reduces the number of groups, and therefore simplifies the iterative grouping performed in the following stage. This initial grouping is followed by an iterative step that progressively combines nearest groups until only two groups remain. The iterative step involves two stages: first, the smallest nonzero entry of the distance matrix is identified. Second, the two groups corresponding to this entry are combined. The distances in the columns, and rows of the first group, are averaged with those of the second group (weighted according to number of isolates in each group). The second group’s rows and columns are then deleted from the distance matrix. The program repeats the iterative step until there are only two groups remaining, creating a phylogenetic “bi-tree” (a tree with one long central branch, and, at either end, a polytomy). A schematic overview of the process, full description of input/output files, and additional features not used in this manuscript are given in File S3.

### Data availability

Reference Data Accession Numbers: Bioproject ID PRJNA299857; Biosample IDs SAMN04216882-SAMN04216894

## Results

### Single nucleotide variation between the 13 Australian strains

Trimmed reads from each resequenced genome were aligned to the reference genome isolate IPO323 v.2.0 using GATK as described in the section *Materials and Methods*. Before quality filtering, over 1 million SNPs were identified between the 13 strains and IPO323. Low quality SNPs (“QD < 2.0 || FS > 50.0 || MQ < 25.0”), and SNPs occurring in “low mappable” (< 0.5 mappability score) regions of the genome were flagged and excluded from further analyses. Many of these SNPs were associated with small structural changes in the genomes, likely larger insertions or deletion events that differ from IPO323, which are often surrounded by a high density of SNP calls. This is also similar for areas near annotated repeat regions, where incorrect mapping of reads to repetitive DNA leads to a high number of SNP calls, and an increase in read mapping coverage. Two examples of SNPs near small indels are shown in Figure S1. Our sequencing coverage ranged from 15x to 30x on average across the genome for most isolates (Figure S2). Due to this limited coverage, and our short size selection (500 bp paired-ends), we did not investigate these potential large rearrangements further.

**Figure 1:**
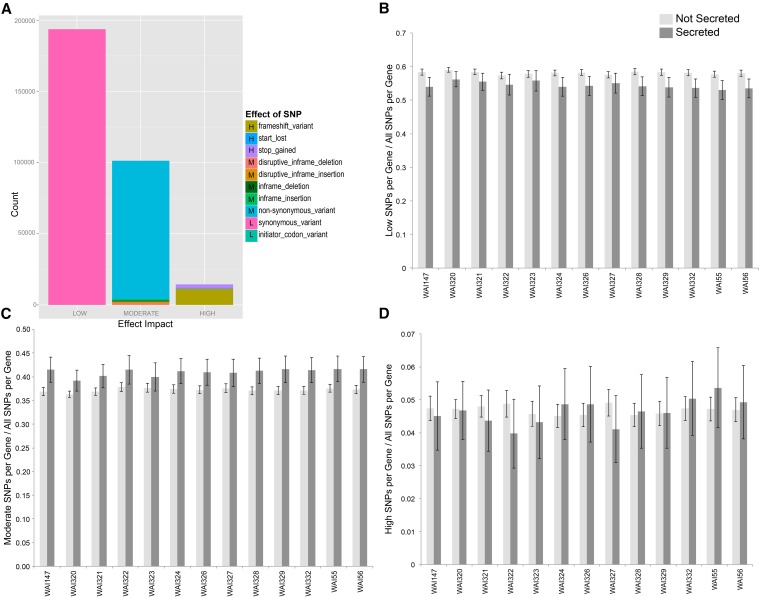
Summary of all single effect SNPs identified in the 13 sequenced genomes using IPO323 as a reference sequence. (A) The number of identified SNPs, colored by their effect on the genome and separated into “Low”, “Moderate,” and “High” effect categories, dependent upon their effect on coding regions. (B) The number of low effect SNPs found within nonsecreted and secreted genes as a percentage of all identified SNPs. The light gray bars show that, on average, Low effect SNPs are found at a higher proportion in nonsecreted genes *vs.* secreted. Black error bars show 95% confidence intervals (CI), from all genes. (C) The number of moderate effect SNPs found within nonsecreted and secreted genes as a percentage of all identified SNPs within a gene. Higher dark gray bars show that, on average, Moderate effect SNPs represent a higher proportion of identified SNPs in secreted genes. (D) There is no observed trend for High effect SNPs in secreted *vs.* nonsecreted genes.

After filtering, the remaining 858,965 SNPs were fed into SnpEff to assess their effect (whether or not they occurred in coding regions) on the genome. SnpEff automatically assigns each SNP into one of four major “impact” categories depending upon the effect that the SNP has on annotated regions of the genome. These categories are: 1) Modifier, SNPs with little to no effect on coding regions (*i.e.*, intergenic). 2) Low, SNPs that occur within coding regions but do not affect the encoded amino acid (*i.e.*, synonymous mutations, introns). 3) Moderate, SNPs that occur within coding regions, and slightly alter the encoded amino acid (*i.e.*, nonsynonymous mutations. 4) High, SNPs that dramatically alter the encoded amino acid (*i.e.*, missense, frame-shift mutations). As expected, most SNPs fell into the Modifier (71.7%) category, followed by Low effect SNPs (17.9%), Moderate (9.0%), and finally High (1.4%). By far the most common SNP location was in intergenic regions, representing 40.3% the total SNPs identified. This was followed by exons (27.4%), downstream (12.2%), upstream (11.9%), introns (7.2%), and 1% in other minor categories. A summary of all single effect SNPs, separated by impact category, for all 13 resequenced isolates is summarized in Figure 1A (12,000 pleiotropic SNPS are excluded from this summary, a complete VCF file is available in File S2). Within coding regions, the most common variant effect was synonymous change (which includes SNPs within introns). This type of SNP was several orders of magnitude higher than High effect SNPs (Figure 1A).

We next considered the distribution of SNPs with different effects between genes with a predicted secretion signal. SignalP v4.1 identified 981 genes with a predicted signal peptide. Figure 1, B–D shows a comparison of the percentage of SNPs within secreted and nonsecreted genes. These results show that a higher percentage of SNPs within nonsecreted genes are classified as Low effect SNPs (*i.e.*, synonymous mutations) (Figure 1B). While this trend is consistent for all resequenced isolates, it was significant [nonoverlapping 95% confidence intervals (95% CIs)] for only eight of the 13 isolates. The reverse trend was seen for the average number of Moderate effect SNPs, with every isolate showing a slight increase in Moderate mutations in genes with a predicted secretion signal (Figure 1C). Again, eight of the 13 isolates had nonoverlapping 95% CI, showing a slight elevation of moderate SNPs in secreted genes. There were no significant differences between any of the isolates between secreted and nonsecreted genes for the High effect SNPs (Figure 1D).

### Presence/absence of coding regions on core and accessory chromosomes

To assess the presence/absence (P/A) of coding genes, each genome was assembled *de novo* to exclude any chance that a gene is called missing due to small rearrangements from the reference. A summary of the final *de novo* assembly statistics for each isolate, including total number of contigs, max contig length, N50 value, and total number of bases assembled (excluding contigs < 500 bp), is provided in Table S1 and Table S2. These statistics are similar to other resequencing projects conducted with *Z. tritici* (Croll *et al.* 2013).

**Figure 2:**
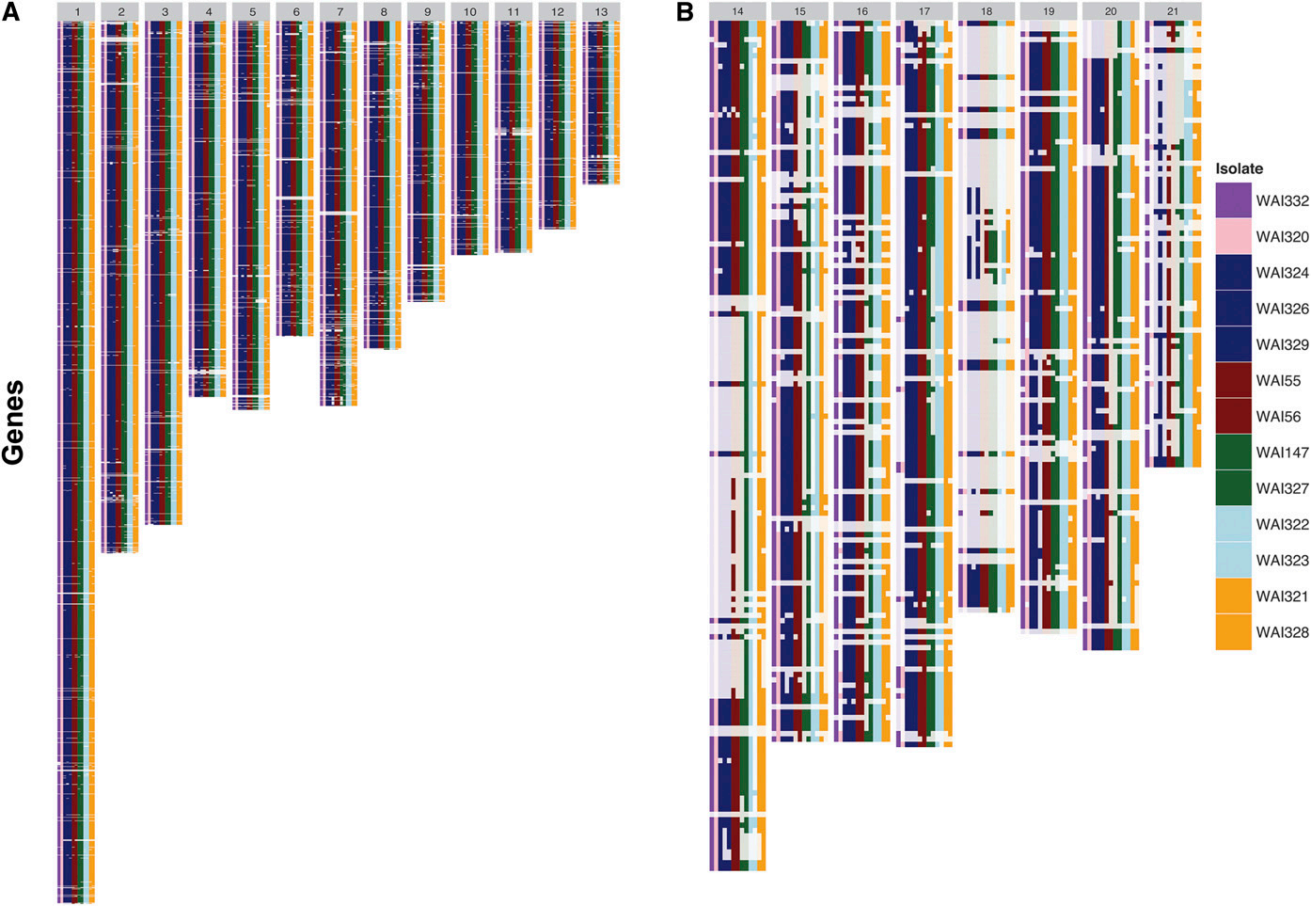
Summary of the presence or absence of IPO323 genes across all chromosomes. Gene presence (dark colors) or absence (translucent) for each re-sequenced isolate is shown for all annotated genes from the reference isolate IPO323. Each row in the figure represents one annotated gene, while each column represents one isolate. There are 13 columns in each panel; the number at the top (in the gray bar) indicates the chromosome. Isolates are ordered from left to right, from earliest to latest year of sampling. Isolates in the same color were sampled during the same year. (A) The core chromosomes of *Z. tritici* possess most of their annotated genes with very few large deletions. (B) The accessory chromosomes possess several large segmental gene deletions, many of which indicate that entire chromosomes are missing or significantly altered in structure from the reference genome.

BLAST searches with the complete coding sequence of each annotated gene were conducted, whereby a gene was called as present if 75% of the gene length was in the top BLAST hit. The complete dataset, which includes a summary by chromosome, is provided in File S4. Figure 2 shows a distinct difference in the pattern of gene P/A between the 13 core chromosomes (CCs) and eight ACs. The isolates, in columns, are ordered from left to right by year of sampling. Some ACs were completely absent in the earlier samples (*e.g.*, Chromosome 14 in isolates WAI332, WAI320, WAI324, WAI329, and WAI329), and appear only in the later columns. The ACs are characterized by large segments of sequential gene presence/absence, whereas on the CCs there were very few instances of large sequential gene absence. Despite many ACs being absent, several genes on these chromosomes were still found in the *de novo* assemblies. Every AC, except for Chromosome 19, was almost completely absent in at least one isolate. While Chromosome 19 was not completely lost in any single isolate, ∼50% of the coding genes were lost in isolate WAI329.

The observation of higher rates of moderate SNPs within secreted genes led us to question whether secreted genes were more likely to be found with P/A polymorphisms. These results are summarized in Table 2; genes that are absent in all Australian isolates are not polymorphic and were not included in this count. Due to the strong difference in gene P/A between core and accessory chromosomes, we summarized the proportion of all or secreted genes for CC and AC chromosomes separately (Table 2). Overall, there was little evidence of elevated rates of gene P/A polymorphisms in secreted *vs.* nonsecreted genes, with the exception of Chromosomes 2 and 10. Large sectional gene absence was far more rare on CCs in comparison to ACs (Figure 2).

**Table 2:** Summary of putative presence/absence (P/A) polymorphisms in secreted and nonsecreted genes by chromosome

Chromosome	# Genes	# Secreted Genes	Proportion Secreted	#Genes in P/A	Proportion Genes P/A	#Secreted Genes P/A	Proportion Secreted PA
CC							
1	1774	163	0.09	135	0.08	13	0.08
2	1069	98	0.09	124	0.12	19	0.19
3	1012	112	0.11	85	0.08	13	0.12
4	755	66	0.09	77	0.10	10	0.15
5	781	80	0.10	88	0.11	12	0.15
6	633	63	0.10	83	0.13	11	0.17
7	774	79	0.10	129	0.17	12	0.15
8	660	56	0.08	68	0.10	4	0.07
9	564	59	0.10	78	0.14	7	0.12
10	470	43	0.09	49	0.10	9	0.21
11	465	50	0.11	62	0.13	5	0.10
12	418	55	0.13	40	0.10	6	0.11
13	328	34	0.10	50	0.15	5	0.15
AC							
14	158	6	0.04	135	0.85	4	0.67
15	134	4	0.03	105	0.78	4	1.00
16	134	3	0.02	97	0.72	2	0.67
17	135	2	0.02	116	0.86	1	0.50
18	110	5	0.02	23	0.21	0	0.00
19	114	0	0.04	56	0.49	0	0.00
20	117	2	0.00	106	0.91	2	1.00
21	83	1	0.02	70	0.84	1	1.00
Total CC	9703	958	0.10	1068	0.11	126	0.13
Total AC	985	23	0.02	708	0.72	14	0.61

CC, Core chromosome; AC, accessory chromosome.

### Linking genes to virulence

**Figure 3:**
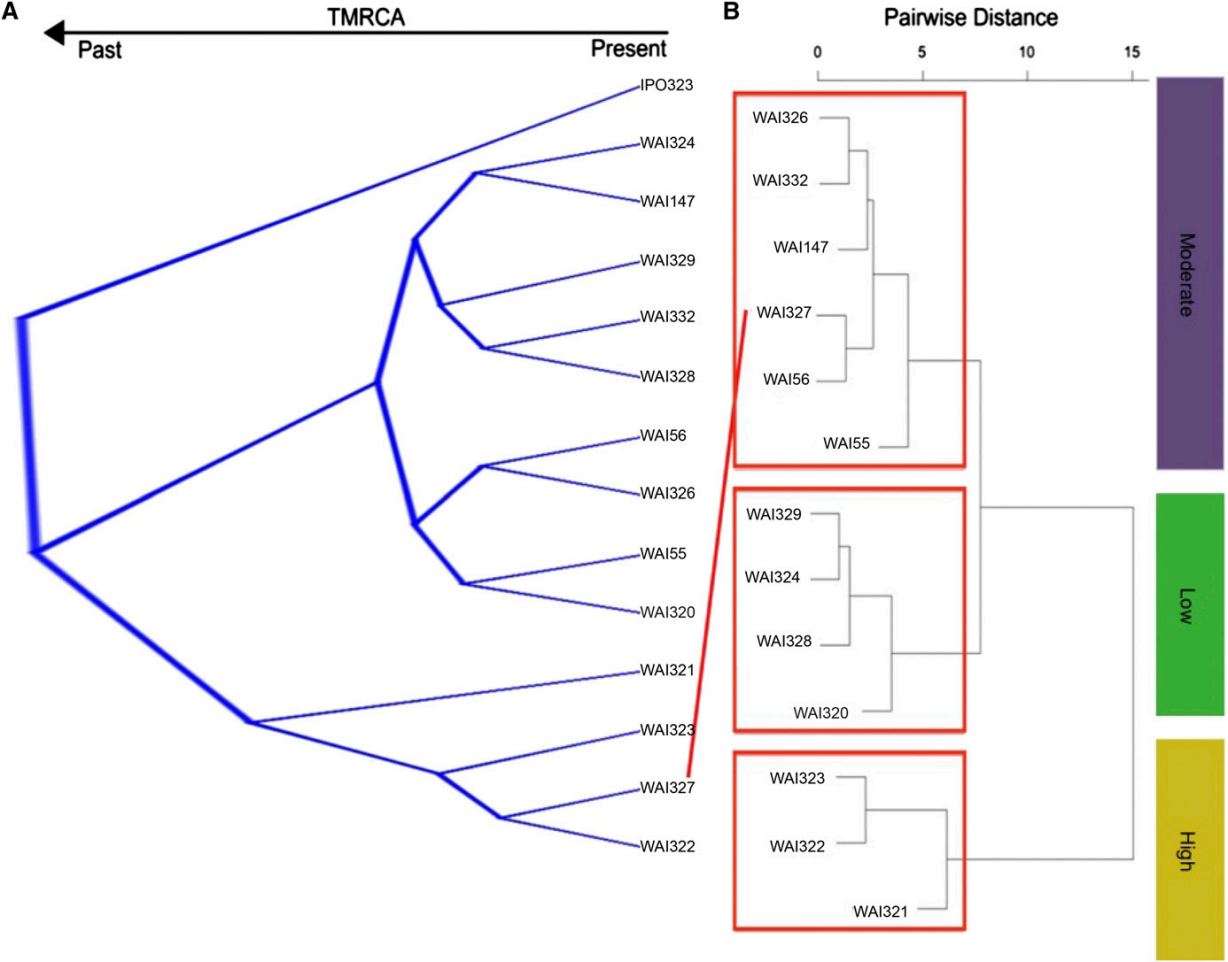
Isolate grouping based on phylogenetic or phenotypic signal. (A) The 10,000 posterior sampled trees estimated with BEAST. Dark blue bars and a lack of crossing over events between branches provides strong support for the topology shown (99.87% of posterior sample). The European reference isolate forms a distinct out-group from the Australian isolates. Two major Australian clades are seen; one with isolates WAI321–WAI323, and isolate WAI327. (B) A cladogram representing the pairwise distances of the Australian isolates based on their qualitative virulence scores. Three distinct groups were identified, which largely match the neutral phylogeny. Isolate WAI327, however, is separated from its closest relatives, clustering more closely with the less virulent isolates.

A Bayesian phylogenetic tree was constructed using 905 aligned genes concatenated into a ∼1.5 Mbp alignment. This analysis was performed three times independently, selecting the genes at random to ensure that gene selection did not dramatically alter the consensus topology from the posterior sample (data not shown). Additionally, the whole genome phylogenetic program andiv0.1 was used to estimate the pairwise distance between isolates based on minimum exact matches throughout the whole genome (Haubold *et al.* 2015). The topology of the consensus tree from the posterior sample, and the tree constructed with pairwise distance estimates from andi, were identical, indicating that concatenation did not bias our Bayesian tree topology. The DensiTree rendering of the posterior sample from Beast is shown in Figure 3A, and the andi dendrogram is provided as Figure S3.

To assess whether the phylogenetic relationship between isolates matched the observed virulence profiles, we performed Ward’s pairwise distance and k-means clustering. The results of k-means clustering, including the curve used to select the ideal number of groups, are presented in Figure S4. The ideal number of clusters should be at the steepest point of the curve, which corresponded to either three or four clusters in our data. Both clustering methods supported the separation of the isolates into three distinct virulence groups. The dendrogram generated with Wards distance clustering is presented in Figure 3B. These three virulence groups can be classified based on their virulence profile into high, medium and low virulence, with the high virulent isolates causing more severe symptoms on a wider range of wheat genotypes (Table 1 and Figure 3B). The neutral phylogeny revealed two highly supported groups of isolates, with WAI321–WAI323, and WAI327 grouped together, distinct from the other sister clade with the remaining nine Australian isolates. The European isolate, IPO323, formed an outgroup from the Australian isolates (Figure 3A). This largely matches the clusters identified by virulence grouping, with one notable exception; isolate WAI327 grouped more closely with the “less-virulent” cluster away from isolates WAI321–WAI323.

The separation of isolate WAI327 from its closest relatives, WAI321–WAI323, suggested that the genes encoding cultivar specificity may be different between these isolates. To pursue this hypothesis, we developed PhyBi (described in *Materials and Methods* and File S3) to identify individual gene trees that group WAI321–WAI323 together without isolate WAI327. PhyBi identified 71 genes out of 10,117 gene alignments that matched this grouping. Of these 71 genes, only six have a signal peptide for secretion. The six secreted genes, along with their location in the genome annotation, and other annotated traits, are listed in Table 3. While the functionality of PhyBi was extended to identify genes that are present or absent from a given gene tree, these results were not summarized, as the analysis showed several hundreds of gene presences/absences throughout the 13 genomes.

**Table 3:** The six secreted genes identified using PhyBi

Gene	Chr.	Length (aa)	SP	JGI ID	InterPro Description	InterPro ID	% Cys	Induced in Kellner *et al.* 2012	Induced in Rudd *et al.* 2015	SNP	dN	dS	Intron	Indel	% Pairwise Identity	% Identical Sites
ZtRRes_00659	1	133	17	NA	NA	NA	0.9	NA	NA	19	3	7	9	0	98.7	96.4
ZtRRes_01714	1	59	20	90262	NA	NA	2.6	No	No	3	2	1	0	0	99.5	98.5
ZtRRes_05182	5	425	21	93433	Major royal jelly	IPR017996	0.2	Yes	Yes	81	5	66	10	26	96.8	92.0
ZtRRes_06114	7	177	19	94274	Extracellular membrane protein, CFEM domain	IPR008427	6.3	No, but highly expressed in all conditions	Highly expressed all days	23	3	11	9	3	98.9	96.4
ZtRRes_08426	10	676	21	96377	NA	NA	3.8	No	No	51	25	19	7	21	98.8	96.1
ZtRRes_08778	11	443	16	111289	NA	NA	2.1	No	No	120	23	86	12	0	96.7	91.3

Chr., chromosome; SNP, single nucleotide polymorphism; NA, not applicable.

**Figure 4:**
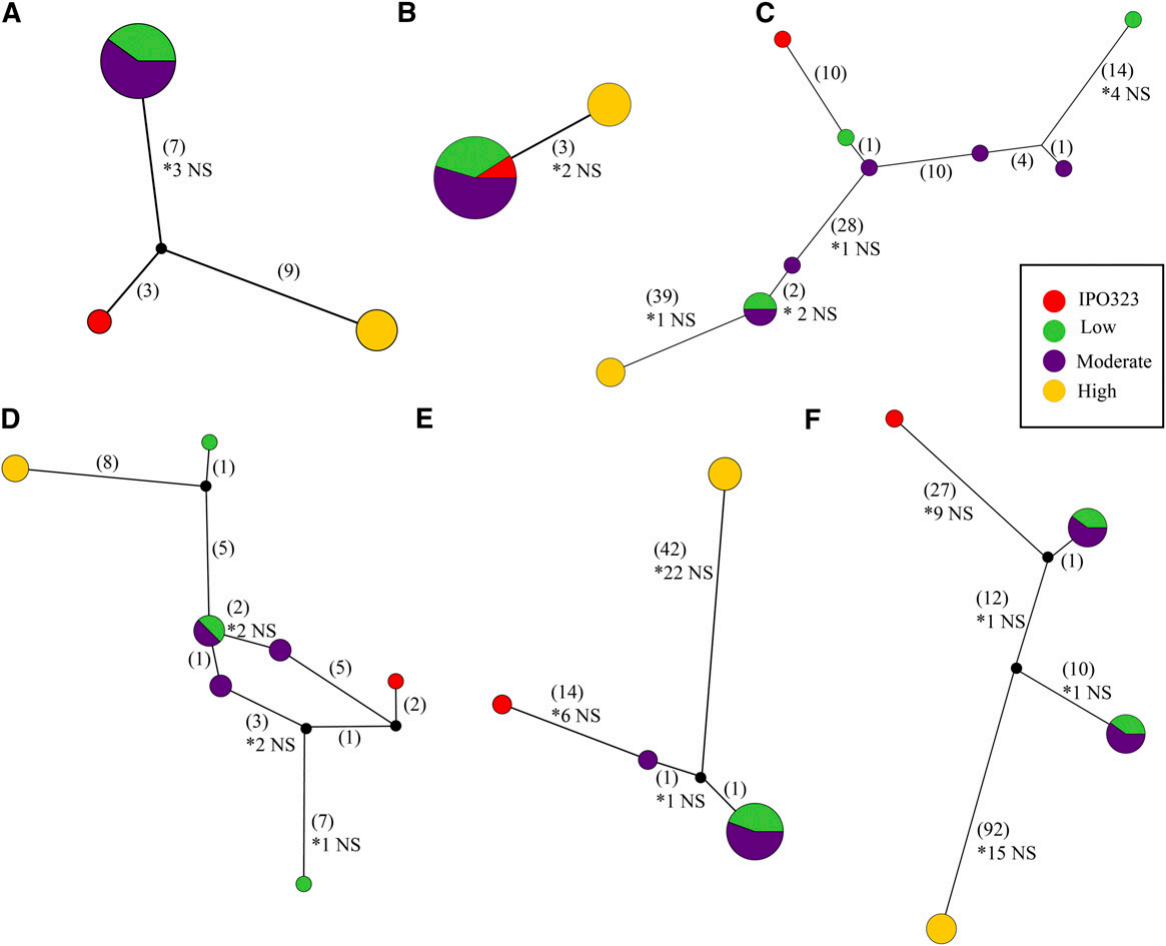
Haplotype networks for six secreted genes identified with PhyBi. Maximum parsimony networks generated with TCS v1.21 and drawn with popart (http://popart.otago.ac.nz). Networks are not drawn to scale and indels were treated as missing data. Single point mutations are shown in parenthesis with the number of nonsynonymous mutations shown with an asterisk below. Haplotype circles are proportional to the number of isolates identified and colored based on their virulence grouping in Figure 3. Black dots are probable unsampled haplotypes as identified by TCS. (A) ZtRRes_00659, (B) ZtRRes_01714, (C) ZtRRes_05182, (D) ZtRRes_06114, (D) ZtRRes_08426, (F) ZtRRes_08778.

The six effector candidates were inspected manually to ensure that intragenic recombination did not interfere with the PhyBi grouping. Five of the six genes matched a reference annotation, while one gene was unique to the Rothamsted annotation (Table 3). Expression of genes whose annotation matched the original genome annotation were compared to two published RNA-seq studies to check for evidence of expression *in planta* (Table 3) (Kellner *et al.* 2014; Rudd *et al.* 2015). None of the six genes were induced during *in planta* infection in either study. Many of these genes matched several characteristics used to classify effector genes (< 300 aa; > 3% cysteine) (Table 3). Three of the six genes encode proteins that are < 250 aa before cleavage of the signal peptide. ZtRRes_01714 is extremely small, and is predicted to be only 39 aa after cleavage of the signal peptide. Three of the identified proteins, however, are large (>400 aa), and would be excluded from traditional effector cutoff values based on their size. One of these large proteins contains a CFEM domain, which has been associated with virulence in *Magnaporthe oryzae* (Dean *et al.* 2005). Haplotype networks were generated for each of the six genes to better visualize the extreme variation in gene sequence between the 13 isolates and IPO323 (Figure 4). All but one of the identified genes has at least one nonsynonymous substitution that separates isolates WAI321–WAI323 from the other haplotypes. Haplotype networks are colored based on each isolates grouping into low, medium, or high virulence phenotype clusters (Figure 3B and Figure 4).

## Discussion

While previous population genetic studies show that the average genetic diversity of *Z. tritici* in Australia is significantly lower than other populations from around the world, this work demonstrates that, across a wider space and time, Australian *Z. tritici* isolates are extremely diverse both genetically and phenotypically (Banke and McDonald 2005; Banke *et al.* 2004; Jürgens *et al.* 2006). This presents a strong challenge to Australian farmers and breeders looking to avoid the impact of this disease, as it has reemerged subsequent to the Millennium Drought. Overall, we identified over 1700 putative gene absences between the reference strain IPO323 and the 13 Australian genomes, and over 800,000 SNPs. For comparison, a resequencing study using 15 isolates of the poplar rust pathogen *Melampsora larici-populina*, with a genome size approximately two times that of *Z. tritici*, identified 611,824 single nucleotide variants (Persoons *et al.* 2014, Duplessis *et al.* 2011; Goodwin *et al.* 2011). These results highlight the extreme diversity that already exists in *Z. tritici*, as well as the challenge of effector gene identification in such a genetic background. In order to further simplify this search, we utilized variation in virulence phenotypes on a range of differential wheat cultivars to target secreted genes, as discussed below.

### Extreme sequence diversity in a limited set of isolate pathotypes

When examining the distribution of Low, Moderate, and High effect SNPs across secreted genes, we noted a significant increase (nonoverlapping 95% confidence intervals) in the number of moderate substitutions (*i.e.*, nonsynonymous substitutions) found in secreted *vs.* nonsecreted genes. While this trend was apparent in all 13 resequenced genomes, it was significant for only eight of the 13 isolates (Figure 1). This data suggests that nonsynonymous substitution is an important means of introducing variation in proteins that are predicted to act outside of the fungal cell. This trend is supported by results from Stukenbrock *et al.* (2011), who also found evidence of higher rates of adaptive evolution in genes with a predicted signal peptide in an interspecific comparison of *Z. tritici*, and its close relatives *Z. pseudotritici* and *Z. ardabiliae*. Recently, some of the genes identified by Stukenbrock *et al.* (2011) were shown to be important for quantitative virulence in seedling infections and host specificity (Poppe *et al.* 2015). There was no difference in the proportion of High effect SNPs found in secreted *vs.* nonsecreted genes. This is an interesting observation, as it implies that, at the genomic scale, introduction of nonsense mutations or frame-shifts is not favored as a means to introduce variation in secreted proteins.

The observed elevation in SNP calls near putative small genome rearrangements within or near annotated genes, suggested that calling the presence or absence genes would be more accurate in *de novo* assemblies (Figure S1). Our previous analysis showed that average read mapping to some of ACs was very low when compared to CCs, and, correspondingly, most of the annotated genes on AC with very low mapping coverage were not found in *de novo* assemblies (Figure 2; McDonald *et al.* 2015). Absence of a chromosome, however, does not also imply loss of the gene annotated on that chromosome. For example, up to 40 out of 110 annotated genes on AC18 are present in *de novo* assemblies, though this chromosome is likely absent, or in a highly divergent form, in these resequenced genomes compared to IPO323. The inheritance of ACs remains a very interesting and perplexing biological phenomenon of *Z. tritici* and its close relatives (Croll *et al.* 2013; Stukenbrock *et al.* 2011).

The virulence data indicates that there are distinct differences in an individual isolate’s ability to infect a particular cultivar. This implies that, within the species, there is further cultivar-specific specialization. Many known effectors with cultivar-specific virulence, such as *Leptosphaeria maculans AvrLm1*, utilize gene deletion as a means of evading host immunity (McDonald *et al.* 2013; Van de Wouw *et al.* 2010). In the case of *AvrLm1*, a simple deletion of the gene renders a plant carrying the resistance gene susceptible to attack. In this study, we postulate that over 1700 genes exist in a presence/absence polymorphism in Australian *Z. tritici* isolates. While this number is likely an overestimation due to misassembly near repetitive regions or telomeres, 1301 out of 1776 putative P/A polymorphisms were found in more than one isolate. Due to the sheer number of potential P/A polymorphisms, it was not possible to link a single gene absence to virulence on a particular cultivar. Larger sequential deletions (not necessarily polymorphic ones) are rare on CCs, but very common on ACs (Figure 2). Importantly, some of the larger segmental deletions, such as the cluster on Chromosome 4 and Chromosome 11, are polymorphic, meaning that sexual recombination can bring this gene deletion in or out of different genetic backgrounds. The isolates selected for this study are too spatially and chronologically diverse to examine the frequency of these deletions in more detail; however, it is tempting to speculate how these larger sequential deletions may change in allelic frequency over time within a field population. Again, these results highlight the endemic genetic diversity of this fungus, and highlight its strong adaptive potential.

### Gene tree-phenotype matching as a novel effector prediction method

Previous attempts to identify effector candidates based on the known properties of effector genes described in other fungi are limited by the large number of potential proteins that they identify (Mirzadi Gohari *et al.* 2015; Morais do Amaral *et al.* 2012). Using information from gene-tree alignments, we were able to directly associate gene sequence with virulence phenotypes. This method assumes that differences at the DNA sequence level will influence the function of the proteins they encode and, in turn, influence the observed phenotype. The advantage of such a strategy is that there is no bias on what the effector proteins should “look” like, eliminating the need for arbitrary cutoffs. Using this method, we were able to assess over 10,000 individual gene trees in < 4 min of computational time, resulting in the identification of six candidate genes.

PhyBi was developed after the observation that many of the individual gene alignments possessed highly divergent haplotypes (Figure 4). These alignments are difficult to automatically pick out of the ∼10,000 predicted genes, because their pairwise identity remains quite high (large diversity between haplotypes but very little within). The haplotype networks shown in Figure 4, E and F nicely demonstrate some of the extreme examples of this phenomenon. The maintenance of two highly divergent alleles at the same locus is often an indication of balancing selection. To test this hypothesis, structured populations from a single field would be required to find the allelic frequency of each haplotype. These gene alignments naturally raise the questions why would these divergent forms of genes be maintained (if not pseudo-genes), and why are so few intermediate haplotypes found?

While three out of six candidate genes in Table 4 encode small proteins, none of the six genes fulfill all of the criteria of typical “effectors”. When compared to previously published *in planta* RNA sequencing data, only one out of the six genes was induced during infection (Kellner *et al.* 2014). This fits with the recent observation by Mirzadi Gohari *et al.* (2015) that highly expressed genes do not seem to overlap with virulence quantitative trait loci.

While there are no truly “avirulent” isolates of *Z. tritici*, isolate-specific interactions with particular wheat cultivars are described extensively in the literature (Arraiano and Brown 2006; Brading *et al.* 2002; Chartrain *et al.* 2004). Similarly, the pathotypes described here show distinct differences in virulence toward wheat cultivars carrying the same postulated resistance genes (*i.e.*, Arina and Hereward, Table 1). These differences in isolate virulence also indicate that there remain uncharacterized resistance genes/loci in these varieties (Table 1). WAI321, WAI322, and WAI323 were selected to group against all other isolates based on the phenotype clustering that did not match the neutral tree (Figure 3). Isolate WAI327, which is genetically similar to these three isolates, is less virulent against four cultivars: Hereward, Tadorna, Veranopolis, and WW2449. These four cultivars are postulated to contain the major resistance genes *Stb6* and *15*, *Stb4*, *Stb2*, and *6*, and *Stb11* and *WW2449*, respectively (reviewed in Brown *et al.* 2015; Goodwin 2007). Note, *Stb*2 and *Stb11* have been mapped to the same location on the short arm of chromosome 1B (Brown *et al.* 2015).

While it is tempting to speculate that the six candidate genes may be an unknown effector virulent against *Stb6* or *Stb2/11*, these major genes are present in only two out of the four cultivars. Alternatively, the gene/s responsible for virulence on these four cultivars could be a more general virulence-associated effector. Genetic knockouts of these genes in the isolates used in this study are currently underway to test this hypothesis.

### Concluding remarks

The lack of understanding of how *Z. tritici* invades its host limits our ability to identify and pursue durable resistance strategies. This study highlights the high levels of existing genetic diversity in *Z. tritici* populations within Australia, and the challenge associated with linking virulence to causal genes, where a million SNPs and hundreds of gene P/A polymorphisms obscure meaningful patterns. This work seeks to simplify this search by utilizing established computational tools (BLAST, alignment, and phlyogenetics) to link divergent gene sequences with matching phenotypes. This method is not limited to virulence data alone, and can be applied to a wide variety of phenotypes, or even interesting genes that violate the relationships shown by neutral genetic markers. This method is particularly useful when large numbers of individuals, required by traditional genome-wide association studies, are difficult to obtain.

## Supplementary Material

Supporting Information
