# Supplementary material for: Utilizing Gene Tree Variation to Identify Candidate Effector Genes in Zymoseptoria tritici
Source: G3 (Bethesda). 2016 Jan 29;6(4):779–91. doi: 10.1534/g3.115.025197 (PMC4825649; doi:10.1534/g3.115.025197)
Supplement: Supporting Information [file supp_6_4_779__index.html]

Utilizing Gene Tree Variation to Identify Candidate Effector Genes in Zymoseptoria tritici — Supporting Information 

# Utilizing Gene Tree Variation to Identify Candidate Effector Genes in *Zymoseptoria tritici*

## Supporting Information for McDonald *et al.*, 2016

**Files in this Data Supplement:**

- Figure S1 - Two examples of questionable SNP calls around potential structural rearrangements. (.pdf, 356 KB)
- Figure S2 - Distribution of Read coverage across the genome for each re-?sequenced isolate. (.pdf, 138 KB)
- Figure S3 - Phylogenomic tree constructed with andi. (.pdf, 100 KB)
- Figure S4 - Results of K‐means clustering based on virulence scores on 18 wheat cultivars. (.pdf, 93 KB)
- File S1 - VCF data. (.zip, 98437 KB)
- File S2 - Bed file used to exclude "low mappability" SNPs. (.zip, 5099 KB)
- File S3 - Details and description of PhyBi. (.doc, 195 KB)
- File S4 - Presence Absence Data. (.xlsx, 1420 KB)
- Table S1 - N50 statistics for *de novo* assemblies using Spades. (.xlsx, 9 KB)
- Table S2 - Isolate details and sampling locations. (.xlsx, 10 KB)
