# Supplementary material for: Utilizing Gene Tree Variation to Identify Candidate Effector Genes in Zymoseptoria tritici
Source: G3 (Bethesda). 2016 Jan 29;6(4):779–91. doi: 10.1534/g3.115.025197 (PMC4825649; doi:10.1534/g3.115.025197)

Figure_S1: Two screen shots from igv showing two isolates (WAI320 and WAI321) aligned to the reference genome IPO323. Blue bars at the bottom are annotated genes. Grey bars are aligned reads and colored lines represent single nucleotide polymorphisms. Note, in one isolate in both pictures there is an excess of SNPs leading up to a small indel. It remains unclear if these SNPs are correct or if the small structural rearrangement has lead to incorrect read mapping.

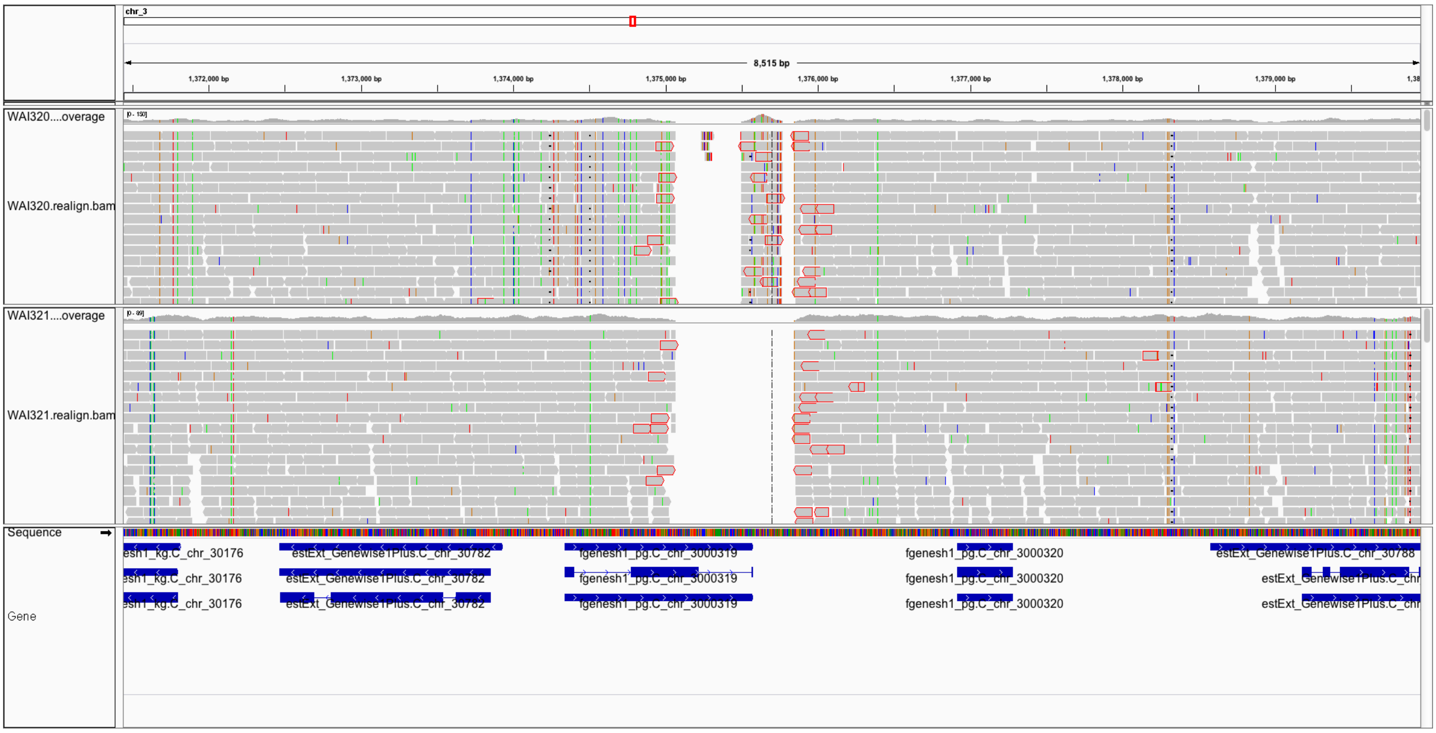

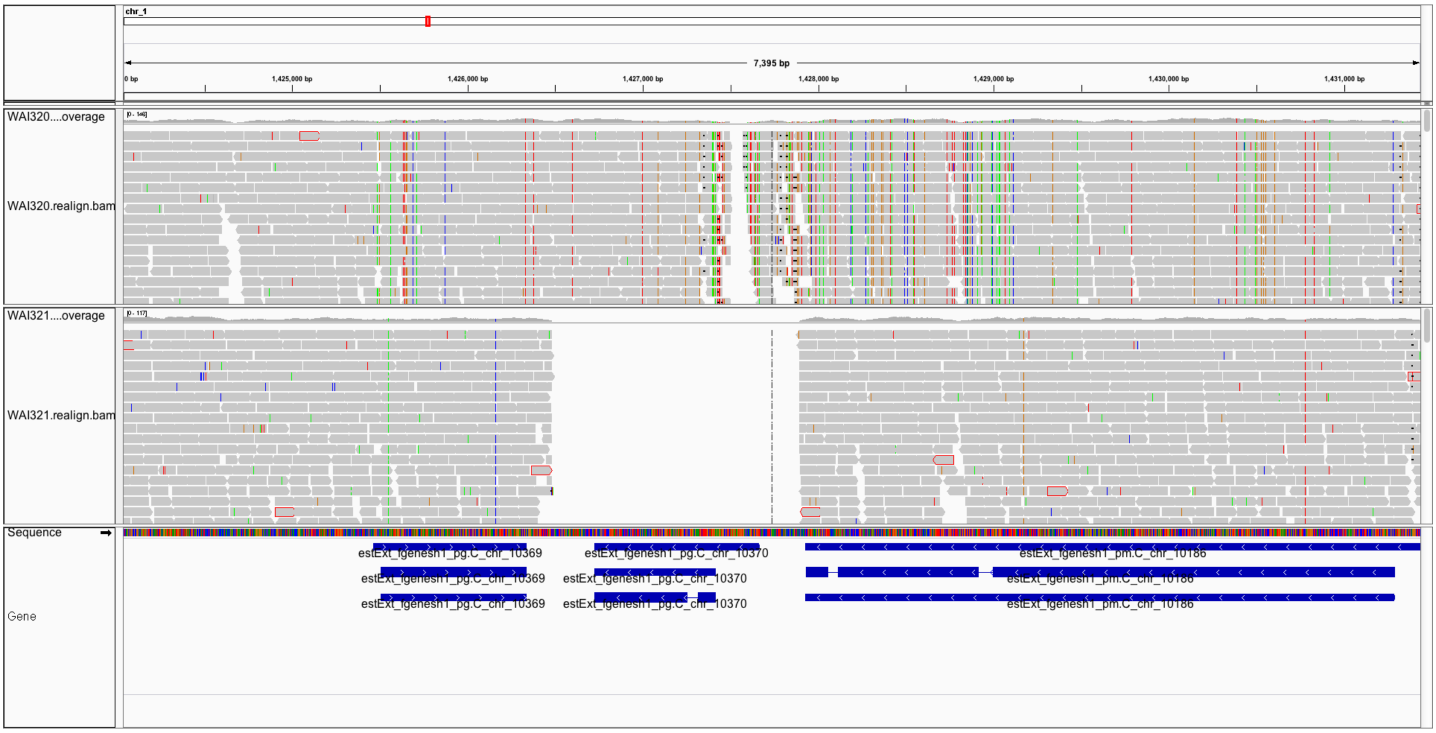

Supplement: Supporting Information [file supp_g3.115.025197_FigureS1.pdf]
