# Supplementary material for: Utilizing Gene Tree Variation to Identify Candidate Effector Genes in Zymoseptoria tritici
Source: G3 (Bethesda). 2016 Jan 29;6(4):779–91. doi: 10.1534/g3.115.025197 (PMC4825649; doi:10.1534/g3.115.025197)
Supplement: Supporting Information [file supp_g3.115.025197_FigureS2.pdf]

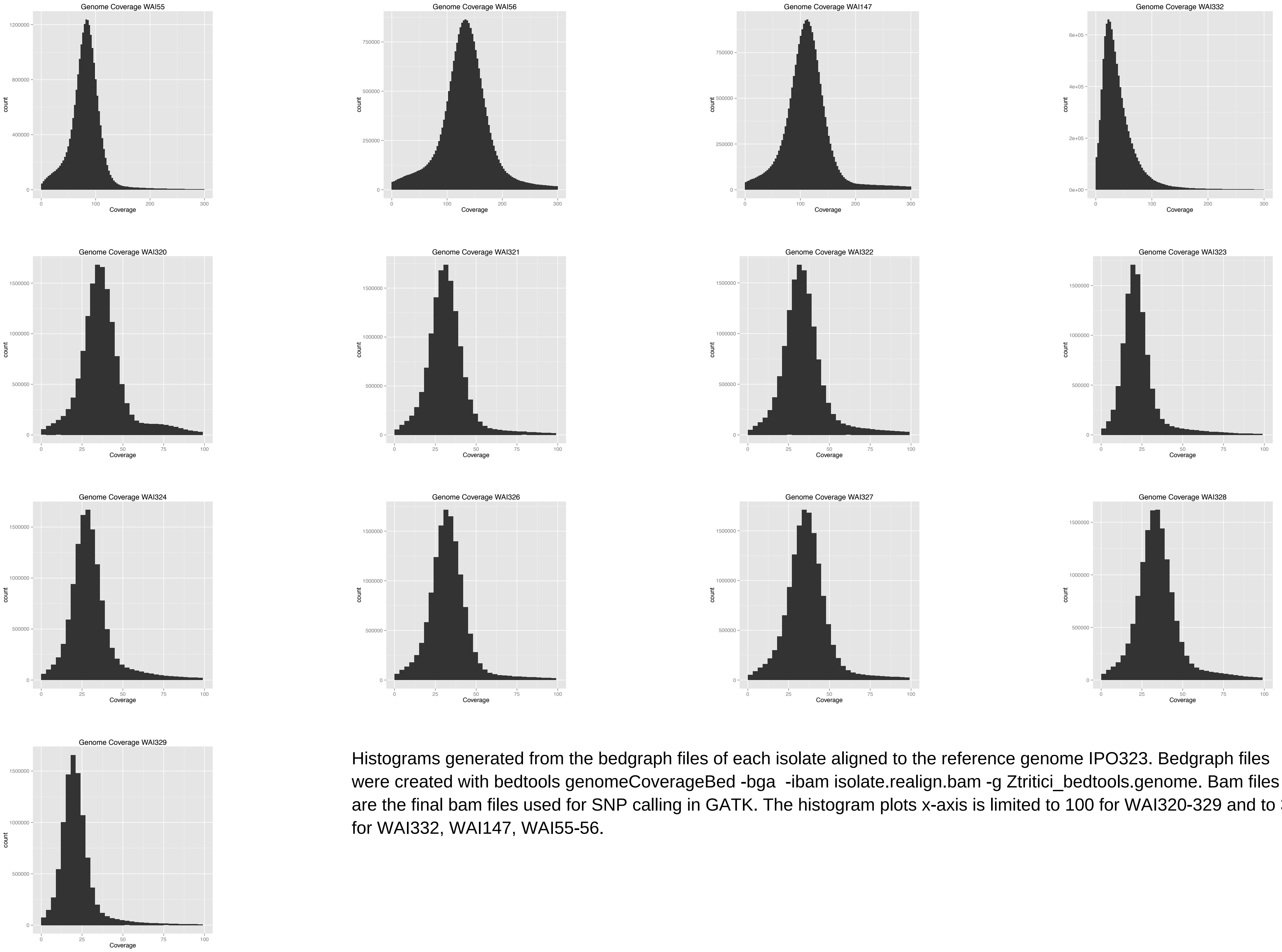

Histograms generated from the bedgraph files of each isolate aligned to the reference genome IPO323. Bedgraph files were created with bedtools genomeCoverageBed -bga -ibam isolate.realign.bam -g Ztritici_bedtools.genome. Bam files are the final bam files used for SNP calling in GATK. The histogram plots x-axis is limited to 100 for WAI320-329 and to 300 for WAI332, WAI147, WAI55-56.
