# Supplementary material for: Utilizing Gene Tree Variation to Identify Candidate Effector Genes in Zymoseptoria tritici
Source: G3 (Bethesda). 2016 Jan 29;6(4):779–91. doi: 10.1534/g3.115.025197 (PMC4825649; doi:10.1534/g3.115.025197)

Figure S3: Dendrogram constructed with the whole genome phylogenetic program andi. Dendrogram was plotted using the distance matrix generated by andi. Input for andi was the entire de novo assembly of each fungal isolate. The topology matches the one constructed using the concatenated gene alignment with BEAST.

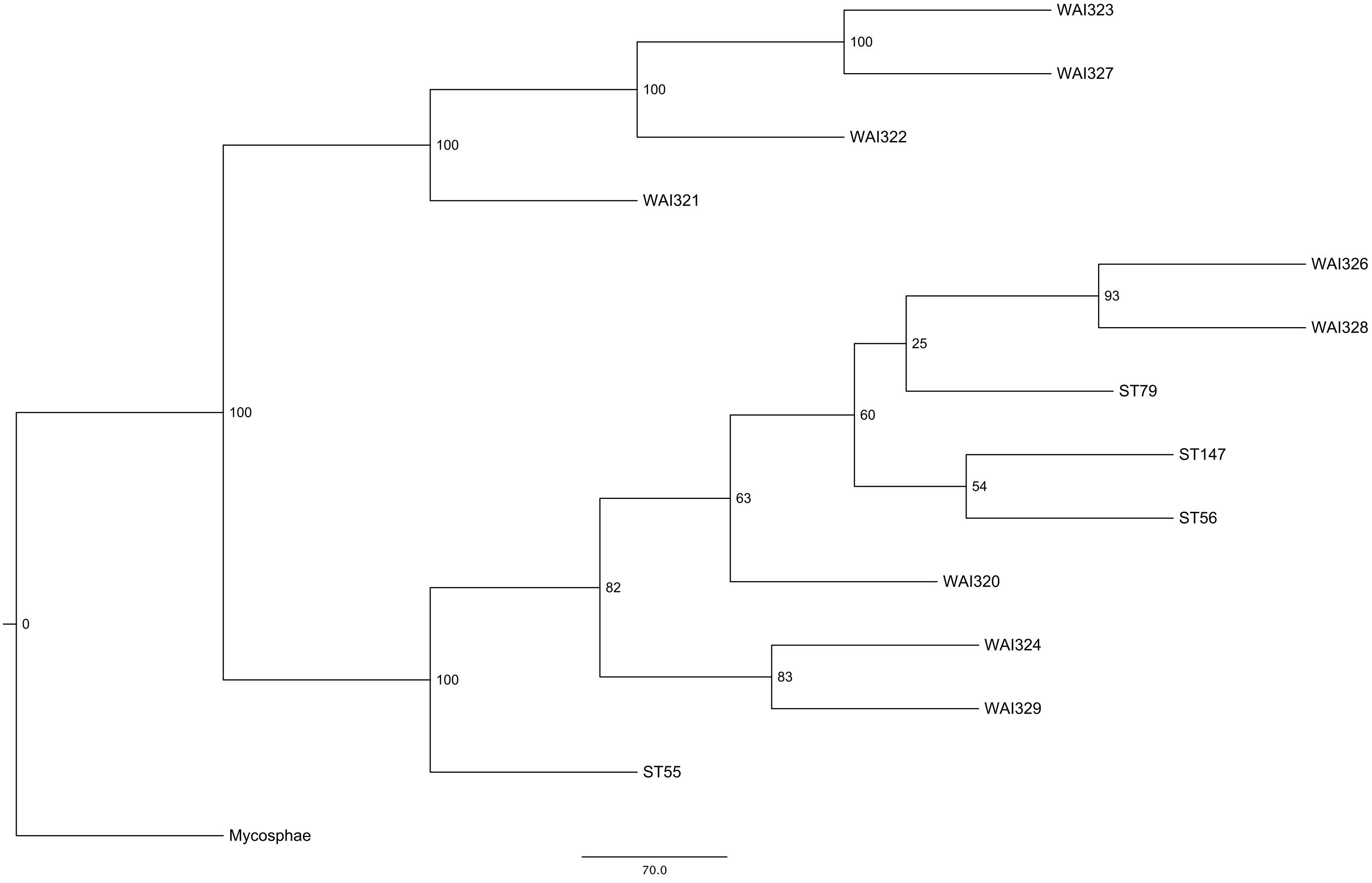

Supplement: Supporting Information [file supp_g3.115.025197_FigureS3.pdf]
