# Supplementary material for: Utilizing Gene Tree Variation to Identify Candidate Effector Genes in Zymoseptoria tritici
Source: G3 (Bethesda). 2016 Jan 29;6(4):779–91. doi: 10.1534/g3.115.025197 (PMC4825649; doi:10.1534/g3.115.025197)

Figure_S4: K-means clustering of virulence data. The top plot shows the kmeans centers (purple dots) surrounded by the isolates who are grouped with that center. Three kmeans clusters were chosen a priori based on the curved line shown in the lower graph.

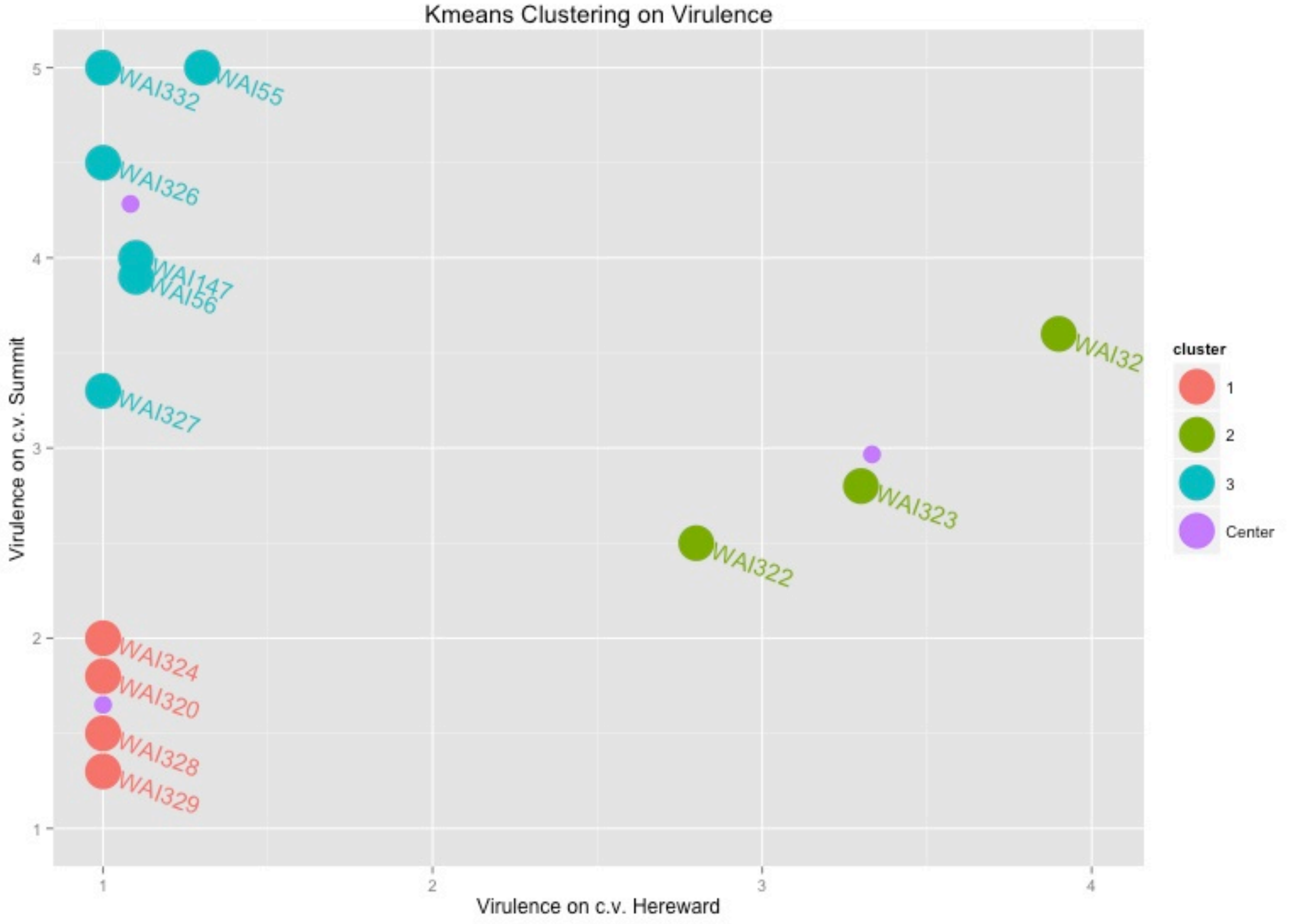

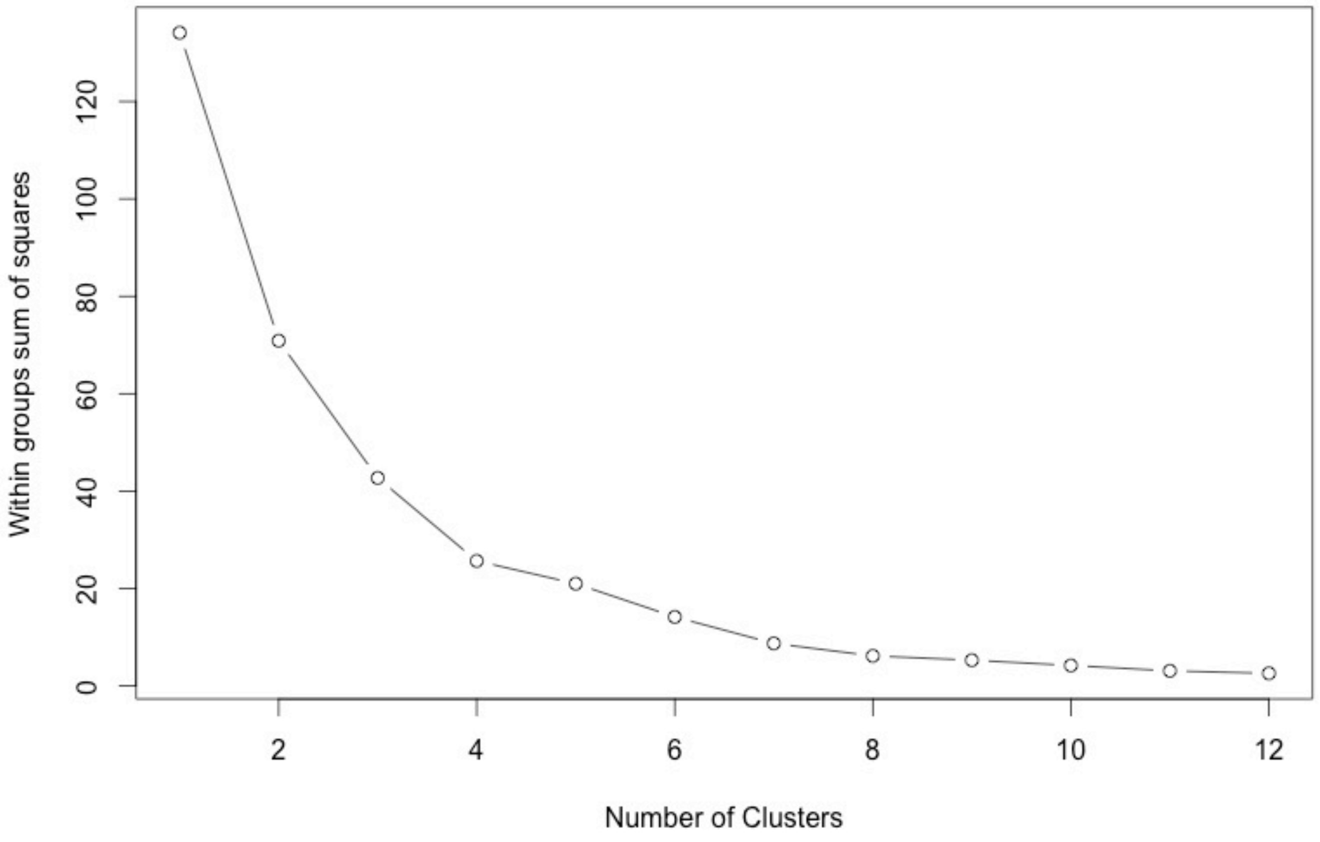

Supplement: Supporting Information [file supp_g3.115.025197_FigureS4.pdf]
