# Supplementary material for: Utilizing Gene Tree Variation to Identify Candidate Effector Genes in Zymoseptoria tritici
Source: G3 (Bethesda). 2016 Jan 29;6(4):779–91. doi: 10.1534/g3.115.025197 (PMC4825649; doi:10.1534/g3.115.025197)
Supplement: Supporting Information [file supp_g3.115.025197_FileS3.doc]

File S3: A schematic overview of PhyBi's newick tree collapse


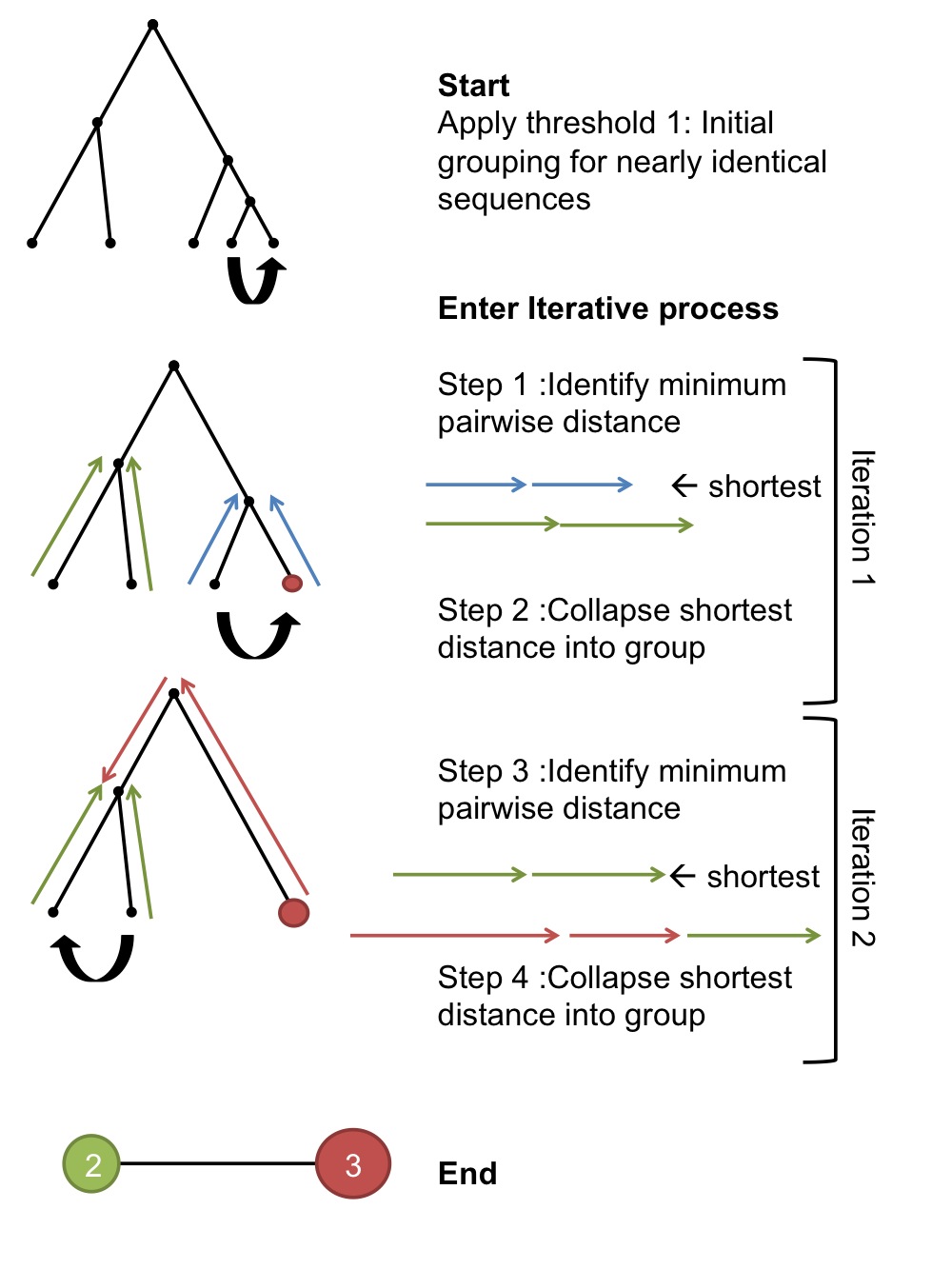


**About Phy-Bi**

Program for collapsing phylogenetic trees into phylogenetic bi-trees.

Phy-Bi requires 3 inputs:

1.) A series of phylogenetic trees in Newick format. The filenames should take the form *.Genename

2.) A .txt file named 'genefiles.txt' which contains the name of each file from 1 on a new line.

3.) A .txt file named 'isolatelist.txt' which contains the name of all isolates to be considered on the first line separated by commas.

Phy-Bi takes the phylogenetic tree corresponding to each gene and splits the isolates into two groups.

This two-group pattern is called a bi-tree. For a given number of isolates there is a limited number of possible bi-trees (combinations). For example if there are 16 possible combinations:

5 combinations which are 4-1

10 combinations which are 3-2

1 combination where all isolates are put into the same group.

If all the isolates are identical this is called a star.

Phy-Bi then creates a file called 'BiTreesSummary.txt' which contains the frequency of occurrence each bi-tree.

#all - AllBin

One of the most useful functions of Phy-Bi is a search function for genes that match a particular bitree pattern (which may correspond to experimental data). If the 'AllBin' function is used then a file is created for each bi-tree pattern in a folder called 'genebins'.

#s- Search

Due to sequencing errors, occasionally genes can be missing from particular isolates. Phy-Bi will ignore missing isolates and continue to operate, however this will lead to a larger number of possible bi-tree combinations. Once the number of of isolates exceeds 6 it becomes impractical to find desired bi-tree combinations by hand. In these cases Phy-Bi has a search function. For search to work the 'isolatelist.txt' input file must contain a second line. This second line is a list of all isolates that the user wishes to be grouped

together.

Bi-Phy will create a file named 'SearchResults.txt' which contains a list of each gene where every present isolate in the second list is sorted into the same group.

#ab - Absence

The absence of certain genes may be of interest to the researcher. If the Absence function is used then Bi-Phy will create a folder called 'AbsentBins', similar to genebins created by the AllBin function. The files created in the AbsentBins folder will contain a list of all possible combinations of absent genes. If both the Search and Absence functions are used then a second list will be created in 'SearchResults.txt' that contains a list of all genes whose absence pattern matches the list inputted for the Search Function. It is recommended that f is set to 0 if Absence is used.

There are a number of other minor functions

#d - Draw

Phy-Bi draws original phylogenetic tree so user can compare output to the input and check that the program is giving reasonable results. Most useful on a small number of genes.

#f - Fraction

The user must give a number between 0 and 1. Phy-Bi will skip genes if too many isolates are missing. This determines the minimum number of isolates that must be present for the gene not to be skipped.

If interested in Absence function, it is recommended that f is set to 0.

#q - Quiet

Silences the default output comments given by Phy-Bi. Avoids cluttering the terminal.

#t - Threshold

Determines the distance required for Phy-Bi to output a star. The default is 0.01, which corresponds to a mutation rate of one in one hundred base pairs

#t2 - Threshold2

A number that indicates how closely related the isolates need to be for Phy-Bi to automatically place them in the same group. The default is 0.0002, which corresponds to a mutation rate of two in ten thousand base pairs.
